# Notes and News

**Published:** 1890-03-01

**Authors:** 


### Notes and News.

Collected and Communicated.

At the annual meeting of subscribers to the Bradford Infirmary and Dispensary, it was stated that a total of 14,000 persons were assisted last year. The debt has been cleared off the building during the year, thanks to the energy shown by the chairman, committee, and the secretary, Mr. Maw. Special wards are now set apart for the treatment of diseases of women, and in every way the Infirmary is marching with the times. The Mayor, who is a firm friend of the institution, presided at the meeting.

The gift of one lakh, or in other words 100,000 rupees, towards the founding of a leper hospital at Bombay by the Hon. Sir Dinshaw Manockjee Petit is the latest of those benefactions which have made the Parsee community of Western India famous throughout the world. This is not the first occasion on which Sir Dinshaw Manockjee Petit has exhibited a noble munificence. During the last twenty-five years Sir Dinshaw has dispensed large sums in public and private charity, principally the latter, and the amount of these benefactions is stated on reliable authority to exceed £200,000. One of the most notable of his latest gifts was to present the freehold of the land on which the Victoria Jubilee Technical Institute has been erected.

The annual report of Charing Cross Hospital is very satisfactory: the medical college is getting on, and the convalescent home has secured a site. Mr. J. B. Martin, at the late meeting, commented on the report, which showed that the number of in-patients for the past year—1,946—exceeded the total for any previous year. The number of out-patients for the year was 21,432, as compared with 19,352. The council regretted that the financial support which the hospital received had not increased in the same proportion. There had in fact, been a falling off in the donations and legacies, though the annual subscription list showed a slight increase—£1,831 as against £1,779 in 1888. The expenditure chargeable to last year amounted to £15,421 1s. 1d. Allowing for the cost of the out-patient department, the cost per bed for the year amounted to £75 15s., or an average of £6 15s. per in-patient. The hospital still maintains its place in the affections of the public, and the wards are always full.

The abstract of expenditure at the Saltburn-by-the-Sea Convalescent Home is curious reading. During 1888 there were 378 patients, and they apparently consumed far more provisions of all kinds than the 475 patients treated last year, or the 673 patients treated in 1887. The cost of a patient for three weeks is in food 19s. 1·39d., and in other expenses 16s. 0·72d.; total, 35s. 2·11d. The Local Board claimed over £100 from the Home in 1888, still that does not cover the extra high expenditure of that year.

Oldham Infirmary in submitting to the public the record of a year's economical management asks increased support from the working classes. Strangely enough in Oldham the Saturday Fund is not nearly so large as it should be. The Lees Annexe has proved most useful, and has been ornamented with engravings given by Mr. MacWhirter, Mr. David Murray, and other artists. The governors, house surgeon, matron, and all the officers of Oldham Infirmary are enthusiastic in their work, and we hope the working classes will soon emulate them.

Some items of news culled from a northern paper are proof positive of how completely it is possible to master a permanent affliction: "Mr. James Paul, the popular deaf-mute missionary, contributes a slashing article to the New York *Deaf-Mutes' Journal*, wherein he denounces the Royal Commission." "Mr. James Macdougall (deaf and dumb), Cambuslang, has been elected captain of Glasgow Deaf Mute Football Club." "Mr. Alexander M. Duff (deaf and dumb) lectured on Magna Charta and Runnymede to the members of the mission in Renfrew Street last week." "Mr. Francis Maginn, the American taught mute missionary of the deaf and dumb, of Belfast, has been laid up with 'la grippe,' but writes to assure his friends of a speedy recovery." These matter-of-fact paragraphs should inspire courage in those similarly afflicted, and should rouse the sympathy and admiration of our readers.

The most important document of this United States Legislative session, considered from a humanitarian standpoint, is the first annual report of the State Commission in Lunacy. The chief part of the report relates to State and county care of the insane. It states that of the nineteen counties now permitted legally to care for their chronic insane few bestow proper treatment upon their inmates, while many present "lamentable instances of squalor, wretchedness and neglect." In no one of these counties are the insane, in any proper sense, under the control of a medical officer. Only two have resident physicians. In only four or five are daily visits of a physician required by law. None of the superintendents of the poor, or keepers, are in the slightest sense qualified for their positions. It is a common practice to bathe several patients in the same water. This has been done in spite of the fact that most lunatics suffer from skin disease—many having loathsome ulcers. The ignorance of the keepers is shown by the fact that none of them seem to disapprove of this shameful and horrible practice. No attention whatever is given to the dietary of patients, that important matter being left usually to an ignorant cook. The number of attendants is in every case too small. The danger of night fires is very great. Little if any attention is given to proper exercise. In few places is reading matter, amusement, or religious instruction provided for, at least in any adequate and proper degree. In many instances the sexes are not separated properly. It is recommended in conclusion that all the insane in the poorhouses of counties other than New York and Kings shall be transferred at the earliest practicable date to State asylums. Considerable excitement has been roused in the States by these disclosures.

THE following vacancies are announced this week, the amount of the salary in each case being given after the name of the institution:

**Resident Medical Officers.**

West London Hospital, House Surgeon—Board, etc.

Addenbrooke's Hospital, House Surgeon—£65, board, etc.

Kent County Asylum, Third Assistant Medical Officer—£120, rooms, etc.

Croydon Union, Assistant Medical Officer and Dispenser—£125, rooms, etc.

Durness, Sutherland, Medical Officer—£150, rooms, etc.

Wolverhampton Eye Infirmary. Resident Assistant—Board, etc.

**Non-Resident Medical Officers.**

Worcester, Medical Officer of Health—£600.

Owen's College, Manchester, Lecturer on Diseases of the Larynx.

London Hospital Medical College, Two Demonstrators of Anatomy, £90.

Victoria Park Hospital for Chest Diseases, Assistant Physician.

King's College, London, Professor of Physiology.

County of Lancaster, Medical Officer—£800.

---

BRADFORD Eye and Ear Hospital is one of those quiet-going institutions, ever progressing slowly and steadily, and ever increasing in public favour. There are some hospitals which are never happy unless they can cry out that they are terribly in debt, or can moan over the necessity for further accommodation or the decrease of subscriptions; but we are not sure that the quieter institutions do not secure more steady support. Bradford Eye and Ear Hospital treated 3,755 patients last year, and managed to pay over £300 to the investment account.

---

A REVIVAL of the report that old horses are exported from this country to Antwerp for the purpose of being turned into extract of beef having taken place, we are requested to state that the Royal Society for the Prevention of Cruelty to Animals recently instituted an investigation into the matter. The society reported that the horses exported from this country were not turned into extract of beef in Antwerp, and that there were no factories in that city where such extract is prepared, but that there were large numbers of old horses exported to Antwerp and legally sold for "horsebeef" in the shops.

---

AN outbreak of puerperal fever in malignant form in the wards of the Philadelphia Lying-in Charity has compelled the managers to close its doors. Dr. Wilson, the physician-in-charge, says the outbreak is the result of a combination of circumstances, chief among which is the fact that the walls of the hospital were not glazed, nor the floors painted, owing to a lack of funds. The hospital will be thoroughly overhauled, disinfected, painted, and varnished, and the floors caulked, and in order to do this the hospital will be closed for one month. During the past 12 months, the hospital has cared for 225 poor patients in its wards, and it has also provided medical attendance, trained nurses, and nourishment for over 300 women in homes other than the hospital. The most rigid asceptic conditions have been carried out in the care of the charity's patients, but this outbreak is undoubtedly caused by the great number of women we have had to care for, coupled with the want of means to extend the plant of the hospital and the bad atmospheric condition, which is largely due to the prevalence of the influenza. Since the outbreak of the disease, one patient has died from sepsis, and one from pneumonia. The winter has been extremely mild, quite like an English one, the thermometer varying from 40 deg. to 70 deg.; two nights it has been as low as 20 deg. There has been but one slight fall of snow, and no ice, and as there is but little prospect of sufficient forming on the ponds so late in the season to allow of its being cut for summer use, there is a prospect of an ice famine, as there are no factories for the manufacture of ice.

ATTENTION has lately been called to the fact that only a fourth of the ladies who went up for the last matriculation at the London University passed. This is held to show that now the usual run of women are going up, and not the pick of their sex, their failures are likely to be as numerous, or more so, than those of the men. Further proof must be forthcoming on this subject though, and for the present, the ladies can console themselves with the success of Miss Annie Mary S. Anderson, of the London School of Medicine for Women, who heads the 1st division of the Intermediate Examination in medicine. The only woman to pass this extremely stiff examination, she deserves praise for standing so high.

---

WE believe we shall do a service in calling the attention of secretaries and officers of hospitals, especially those connected with the smaller charities, to the system of the National Safe Deposit Company, at Queen Victoria Street, Mansion House, E.C. They would find here in a central position absolute security for any money or other valuables which they may find it necessary to keep during the week, or from time to time, and anyone can inspect the elaborate system of safes and strong-boxes on making application to the secretary. A visit to this steel-clad fortress cannot fail to interest and instruct, if it does not determine the visitor to make use of the facilities here afforded to all who have valuables, either of their own, or in trust for others.

---

WE observe a letter signed Charles Mansfield Owen in the *Morning Post*, headed "Teaching the Dumb to Speak." Somebody else wrote that teaching the dumb to speak had completely died out in England between 1760 and 1863, and implied that to Mr. Van Praagh belongs the honour of its revivification. But Mr. Owen says those who regard Mr. Van Praagh as the pioneer and original instructor have but a superficial knowledge of the subject, for teaching of speech has always proved a feature in the education of the London Asylum from its foundation in 1792. Moreover, Mr. Owen asserts that the oral method in this country has proved a failure. Into these matters of controversy we do not propose to enter. But we note that Mr. Owen says something about the "hundreds of men and women now *teaching* the system in England." We have printed the word "teaching" in italics because if there are hundreds teaching there must be many hundreds learning. We cannot at this moment lay hands on any reliable statistics as to the number of deaf and dumb persons in proportion to the whole population, but it must be enormous, and, moreover, there is reason to believe it is increasing. Some facts were brought to our notice the other day which it may be as well to detail. A gentleman having occasion to call at a small house in a London suburb, noticed a fine-looking little boy sitting at the table. "Yes," said his grandmother, "he's a fine little boy, but he's deaf and dumb." Then the gentleman of course made some sympathising remark. "Oh!" said the old grandmother, "he's not the only only one deaf and dumb; there are two others in the asylum, and he's going when he's old enough; and there's a deaf and dumb baby, too!" On inquiry it appeared that neither the father nor mother was afflicted in a similar manner. But the gentleman, not being a medical man, made no further investigation into the family history. Now it is well known that dumbness may occur from many causes. But the most frequent cause is congenital or early complete deafness, so that the person is unable to acquire the knowledge of articulate sounds. The calamity must be regarded as, like many other maladies, more or less hereditary. What can be said, therefore, in favour of marriage between deaf and dumb persons? Absolutely nothing.

